# Expression of dsRNA in recombinant *Isaria fumosorosea* strain targets the *TLR7* gene in *Bemisia tabaci*

**DOI:** 10.1186/s12896-015-0170-8

**Published:** 2015-07-22

**Authors:** Xiurun Chen, Lin Li, Qiongbo Hu, Bowen Zhang, Wei Wu, Fengliang Jin, Junxi Jiang

**Affiliations:** College of Agriculture, South China Agricultural University, Guangzhou, China; College of Agronamy, Jiangxi Agricultural University, Nanchang, China

**Keywords:** *Isaria fumosorosea*, Recombinant strain, dsRNA, Whitefly

## Abstract

**Background:**

RNA interference (RNAi) technology shows a great potential in controlling agricultural pests, despite the difficulty of introducing exogenous dsRNA/siRNA into target pests. *Isaria fumosorosea* is a common fungal pathogen of the B-biotype *Bemisia tabaci* (whitefly), which is a widespread pest. Entomopathogenic fungi directly penetrate the cuticle and invade insect hemocoel. Application of *I. fumosorosea* expressing dsRNA of whitefly immunity-related gene may aid in developing RNAi technology to effectively control whiteflies.

**Methods:**

A dsRNA expression plasmid, psTLR7, was constructed by introducing the Toll-like receptor 7 (TLR7) gene of B-biotype whitefly to the silent vector, pSilent-1. The plasmid psTLR7 was transferred into the protoplast of the I. fumosorosea strain IfB01. Then, the recombinant strain was screened out based on the biological stability and bioactivity against whitefly.

**Results:**

A genetically stable recombinant strain IfB01-TRL7 was screened out. The impact of IfB01-TRL7 against whitefly TRL7 gene was validated by qPCR. Lower expression levels of the TLR7 gene was observed in the whiteflies infected by the recombinant strain. The bioassay results indicated that compared to IfB01 strain, IfB01-TRL7 increased the mortality of whitefly nymphs, and decreased and shortened the values of LC_50_ and LT_50_, thus indicating higher virulence of IfB01-TRL7.

**Conclusion:**

The expression of the dsRNA of whitefly *TLR7* gene in recombinant *I. fumosorosea* strain successfully knocked down the host target gene by infecting the nymphs and enhanced the whiteflies mortality. The present study will give insight to new application of RNAi technology for more effective biocontrol of this pests.

**Electronic supplementary material:**

The online version of this article (doi:10.1186/s12896-015-0170-8) contains supplementary material, which is available to authorized users.

## Background

*Bemisia tabaci*, commonly known as the whitefly, is an important species complex, and its B-biotype has been named as a superbug [1, 2]. Whiteflies not only directly affect the growth and development of host plants by sucking sap, but also seriously injure the crop production by spreading begomoviruses, such as Tomato yellow leaf curl virus (TYLCV) and Tomato yellow leaf curl China virus (TYLCCNV) [3–5]. At present, chemical insecticides remain the main mode of pest control. However, the serious problems associated with the increasing use of chemicals, such as environmental pollution, food security, and poisoning, demand an urgent need for new alternative technologies [3].

RNA interference (RNAi) is considered to be a novel technology to control agricultural pests [4–7]. However, the difficulty of introducing exogenous dsRNA/siRNA into target pests blocks its application in fields. To date, the common dsRNA/siRNA delivery methods, including injection and oral feeding, are mainly used under laboratory conditions, and only a few transgenic host plants have been used in the fields after safety evaluation [7–9]. Several researchers have reported about the whitefly RNAi. In one such study, the injection of dsRNA molecules (directed specifically towards genes uniquely expressed in the midgut and salivary glands) into the body cavity of whiteflies caused 70 % reduction in the gene expression levels as compared to the whiteflies injected with buffer or with a green fluorescent protein (GFP)-specific dsRNA [10]. By oral route, the ds/siRNAs of actin ortholog, ADP/ATP translocase, alpha-tubulin, ribosomal protein L9 (RPL9), and V-ATPase A subunit caused 29–97 % mortality in the whitefly [11]. Recently, transgenic tobacco lines were developed for the expression of long dsRNA precursors of siRNA and for knocking down the V-ATPase A mRNA in whiteflies. When these pests fed on the transgenic plants, their transcript level of V-ATPase A was reduced down to 62 % [12]. In our previous studies, the dsRNAs of five genes (TLR7, GNBP1, integrin alpha-PS1-like isoform 3, C-type lectin-like precursor and phenoloxidase subunit A3-like) related to innate immunity were delivered into whitefly adults through oral and contact treatments. The 68–96 % reduction of gene expressions and 71–97 % mortalities of whitefly adults were yielded in the oral treatments, at a dosage of 100 μg/mL. Among them, the *Toll-like receptor 7* (*TLR7*) was found to be the most susceptible gene. However, the contact treatments failed to deliver any impact (Hu et al., unpublished data). These findings suggest that RNAi technology can be further developed to control the spread of whiteflies.

Entomopathogenic fungi are often used as myco-insecticides to control sucking sap pests. Entomogenous fungi mainly invade insects through the cuticle and morphologically change into blastospores after entering the host’s hemocoel to adapt the hemolymph immunity [13, 14]. Toll pathway plays important role in defending fungal infection. The induction of the Toll pathway by fungi leads to the activation of cellular immunity as well as the systemic production of certain antimicrobial peptides. The Toll receptors are essential for immunity. They are activated when the proteolytically cleaved ligand Spaetzle binds to the receptors, eventually leading to the activation of the NF-kB factors Dorsal-related immunity factor or Dorsal. In total, nine Toll receptors are encoded in the Drosophila genome [15, 16]. However, in Drosophila, Toll-7 is considered an antiviral gene for pattern recognition receptors of vesicular stomatitis virus [17]. Many Toll-like receptors (TLRs) were discovered in other insects, but their exact functions are not clear yet [18, 19].

*Isaria fumosorosea* is a species complex of entomopathogenic fungus with wide geographical distribution and extensive range of host insects, including the orders, Hemiptera, Lepidoptera, Diptera, Coleoptera, and Hymenoptera [20, 21]. *I. fumosorosea* is also the common fungal species infecting the whitefly nymphs. In a previous research, we screened an *I. fumosorosea* strain, IfB01, which performed as a good control against whiteflies [22]. With the advent of gene modification techniques in entomopathogenic fungus [23–25] and the development of RNAi technology, the present study aimed at constructing a recombinant strain, which expresses dsRNA to knock down the whitefly immune-related gene, TLR7, so as to improve the insecticidal activity of the fungal strain.

## Materials and methods

### Rearing of B-biotype *B. tabaci*

The B-biotype *B. tabaci* population used in this experiment was reared for three years and more than twenty generations in a greenhouse with the host plant, *Hibiscus rosasinensis* Linn. During the bioassay, the adults of whiteflies were moved onto the pot-planted *H. rosasinensis* for 24 h for laying eggs. Subsequently, the pot plants with eggs were cultured in an incubator at 25 °C, RH 70 %, and under a photoperiod of 12 L:12D.

### Culturing of *I. fumosorosea* strain, IfB01

The *I. fumosorosea* strain, IfB01, was used as the parent strain. IfB01 was isolated by our research group and reserved at the China Centre for Type Culture Collection (CCTCC, Wuhan, China), with an accession number, M2012800. The slant of IfB01 strain was inoculated on Czapek-Dox plate (peptone, 5 g; NaNO_3_, 3 g; K_2_HPO_4_, 1 g; MgSO_4_ · 7H_2_O, 0.5 g; KCl, 0.5 g; FeSO_4_ · 7H_2_O, 0.01 g; sucrose, 30 g; agar, 15–20 g; ddH_2_O, 1000 mL) and incubated at 25 °C for two weeks. The conidia were subsequently collected from the plates and suspended with 0.05 % Tween 80 to attain a concentration of 10^8^ spores/mL. For liquid culture, 1 mL conidial suspension was inoculated into 100 mL Czapek Dox broth and cultured at 25 °C, 150 rpm for 30 h to collect the mycelia for preparation of the protoplast.

### Construction of recombinant plasmid

The total RNA from the adult B-biotype of *B. tabaci* was extracted using Trizol Total RNA Isolation Kit (Takara, Japan) according to the manufacturer’s protocol. The cDNA was synthesized using the M-MLV Reverse Transcriptase (Takara, Japan) following the manufacturer’s protocol. The primers, TLR7F/TLR7R, were designed based on the whitefly *TLR7* gene sequence as the target gene (from transcriptome unigene sequence, Unigene20701_B4592195; Additional file 1). The PCR products were detected using 0.8 % agarose gel electrophoresis, and sequenced at the Guangzhou Yingjun Biotechnological Company (Guangzhou, China).

The PCR primers, TLR7F-1/TLR7R-1 and TLR7F-2/TLR7R-2 (Table 1), with restriction enzyme sites were used to the forward and reverse ends of the target fragment. The expected size of the target fragment was 548 bp (Additional file 2). The PCR amplification was performed as following: pre-denaturation at 94 °C for 4 min; 35 cycles of denaturation at 94 °C for 1 min, annealing at 56 °C for 30 s, extension at 72 °C for 45 s; and a final extension at 72 °C for 10 min. PCR products were separated on 0.8 % agarose gels and visualized by ethidium bromide staining. The forward and reverse fragments of the target gene were recovered and confirmed by electrophoresis and sequencing.

**Table 1:** List of primers used in PCR

Name	Sequence	Used for
TLR7F	5′-CAC GCC GAA AGT TTC ATC TA-3′	cDNA PCR
TLR7R	5′-TTG TCG TTC AAA AGG AGGG-3′	cDNA PCR
TLR7F-1	5′-CCG CTCGAG CAC GCC GAA AGT TTC ATC TA-3′ XhoI	Forward fragment PCR
TLR7R-1	5′-AGC AAGCTT TTG TCG TTC AAA AGG AGGG-3′ HindIII	Forward fragment PCR
TLR7F-2	5′-CGG GGTACC CAC GCC GAA AGT TTC ATC TA-3′ KpnI	Reverse fragment PCR
TLR7R-2	5′-CAT GCATGC TTG TCG TTC AAA AGG AGGG-3′ SphI	Reverse fragment PCR
HygB-F	5′-CGA CAG CGT CTC CGA CCTGA-3′	PCR of hygromycin B phosphotransferase gene
HygB-R	5′-TTC GAT GAT GCA GCT TGG GCG-3′	PCR of hygromycin B phosphotransferase gene
TLR7-VF	5′-GTCACCGACGAAATCC-3′	Validation for expression of TLR7 gene of whitefly
TLR7-VR	5′-AGAGTCCCAGCCTTGTT-3′	Validation for expression of TLR7 gene of whitefly
β-actin-F	5′-TCACCACCACAGCTGAGAGA-3′	Validation for expression of TLR7 gene of whitefly
β-actin-R	5′-CTCGTGGATACCGCAAGATT-3′	Validation for expression of TLR7 gene of whitefly

The vector, pSilent-1, was kindly provided by the Fungal Genetic Store Center of America (University of Missouri, Kansas City, MO, USA).

The forward fragment and plasmid pSilent-1 were digested with the double enzymes, *Xho*I and *Hind*III, respectively. After purifying, they were linked with T4 ligase enzyme, incubated overnight at 4 °C, and then, transformed into competent *E. coli* DH5α cells to form the plasmid, pSilent-1-TLR7F. This plasmid and the reverse fragment were then digested with *Sph*I and *Kpn*I, linked T4 ligase enzyme again, and transformed into *E. coli* DH5α. Finally, the new plasmid was obtained and validated by double enzyme digest with *Xho*I and *Hind*III, and *Sph*I and *Kpn*I.

### Transformation of IfB01 strain

The preparation and regeneration of protoplasts from the IfB01 strain were carried out according to the protocols described by Li et al. [26]. The protoplasts were obtained from 30 h-old mycelia following treatment with 1 % snailase, 2 % cellulose, and 0.7 mol/L NaCl pH 6.0 (an osmotic stabilizer), and incubation at 30 °C and 100 rpm for 8 h. The protoplasts were suspended in 1 mL STC solution (1.2 mol/L Sorbitol, 10 mmol/L Tris–HCl pH 7.5, 50 mmol/L CaCl2; autoclaved at 121 °C for 25 min); then, 10 μL plasmid pSilent-1 was added to the prepared protoplast suspension and mixed carefully. After holding it at 4 °C for 1 h, 200 μL PTC solution (40 % PEG4000, 10 mmol/L Tris–HCl pH 7.5, 50 mmol/L CaCl2, sterilized with 0.45 μm bacteria filter) were added to the mixture and stored at room temperature for 20 min. Subsequently, 800 μL PTC solution was added drop wise for shock culturing at 50 rpm for 20 min, then 5 mL liquid regeneration medium was added [26] to the culture and incubated overnight at 25 °C and 150 rpm. The recombinants were isolated from the plates of 200 μL protoplast regeneration medium [26] containing 200 μg/mL hygromycin B, cultured with the pellets of the aforementioned mixture at 25 °C for 30 h.

### Screening of the recombinant strain

The fungal plaques were picked out and transferred from the regeneration medium plates to PDA plates, and were cultured at 25 °C for a week. The mycelia were then cut with a punch and transplanted into PD broth with Amp (Ampicillin Sodium Salt), and were continually cultured at 25 °C and 150 rpm for 2 days. The mycelia were collected from the broth and used for extracting the recombinant DNA with a DNA Extraction Kit (Bioteke, Beijing, China), using the manufacturer’s protocol. The quality of the extracted DNA was assessed by 0.8 % agarose gel electrophoresis and was later stored at−20 °C. For further PCR validation, the primers TLR7F/TLR7R and HygB-F/HygB-R were used to detect the target fragment and *hygromycin B phosphotransferase* gene (*Hph*^*r*^), respectively, under the same cycling conditions as described earlier. The PCR products were recovered using 0.8 % agarose gel electrophoresis and subsequently sequenced at the Beijing Liuhe Huada Gene Technology Company (Beijing, China). The strains simultaneously containing fragments of *TLR7* and *Hph*^*r*^ genes were determined as recombinant strains. The positive strains were inoculated from the broths to PDA plates, and then, ten plaques were picked out for further screening of genetic stable recombinants. They were subjected to three successive transfers of cultures on PDA plates containing 200 μg/mL hygromycin B; then, transferred into PDA plates for three consecutive cultures; and were later transferred into PDA plates containing 200 μg/mL hygromycin B. After the transferring cultures, the conidia were collected for the PCR detection of target fragment and *Hph*^*r*^ gene. The best stable recombinant was screened and subjected to studying the colony morphology and analyzed for their biological characteristics.

### Bioassay of virulence of IfB01 and IfB01-TRL7 strains against whitefly

The conidia of IfB01 and IfB01-TRL7 strains were collected from Czapek-Dox plates and were suspended in 0.05 % Tween-80 to obtain a final stock concentration of 1 × 10^8^ spores/mL. Further, the working suspensions of concentrations−2 × 10^7^, 1 × 10^7^, 5 × 10^6^, 2.5 × 10^6^, and 1.25 × 10^6^ spores/mL were prepared. The leaf immersion method (China standard NY/T 1154.14-2008) was employed to the bioassay. Before treatment, the potted plants of *H. rosasinensis* were checked to ensure that approximately 100 sec instar nymphs remained on each leaf. During the treatment, the leaf with whitefly nymphs was dipped into each working suspension for 30 s. After drying, the potted plants were cultured at 25 °C under a photoperiod of 14 L:10D. Four leaves were utilized for each treatment. The control treatment used was 0.05 % Tween-80. The experiment was replicated two times. The cumulate mortality at 4, 6, 8, 10, and 12 d post-treatment was investigated. The calibrated mortalities, LC-p, LT-p curves, and the values of LC_50_ and LT_50_ were evaluated by means of SPSS Statistics software version 19 (IBM, USA).

### Validation for expression of *TLR7* gene of whitefly

The qPCR method [27] was used to validate the expression of *TLR7* gene in whitefly. Three cadavers of 3rd–4th instar nymphs infected by IfB01 and IfB01-TRL7 strains were collected for RNA extraction. Three living 3rd–4th instar nymphs were used as controls. The experiment was repeated three times. The primers, TLR7-VF and TLR7-VR (Table 1), were employed for PCR amplification of the *TLR7* gene of whitefly. The *β-actin* gene, considered as an internal reference gene, was amplified with the primers, β-actin-F and β-actin-R (Table 1). The qPCR was performed on Bio-Rad CFX Connect™ Real-time Thermal Cycler (Bio-Rad, USA). The reaction mixture contained target genes DNA template, 1 μL; TLR7-VF, 0.5 μL; TLR7-VR, 0.5 μL; iTaq^TM^ Universal SYBR® Green Supermix, (Bio-Rad, USA), 10 μL; and ddH_2_O, 8 μL. The cycling conditions involved pre-denaturation for 2 min at 94 °C, and 35 cycles of denaturation for 30 s at 94 °C and annealing for 30 s at 51 °C. The expressions of target genes were quantified by evaluating the values of 2^-ΔΔCt^.

## Results

### Construction of dsRNA expression plasmid targeting *TLR7* gene of whitefly

**Fig. 1:**
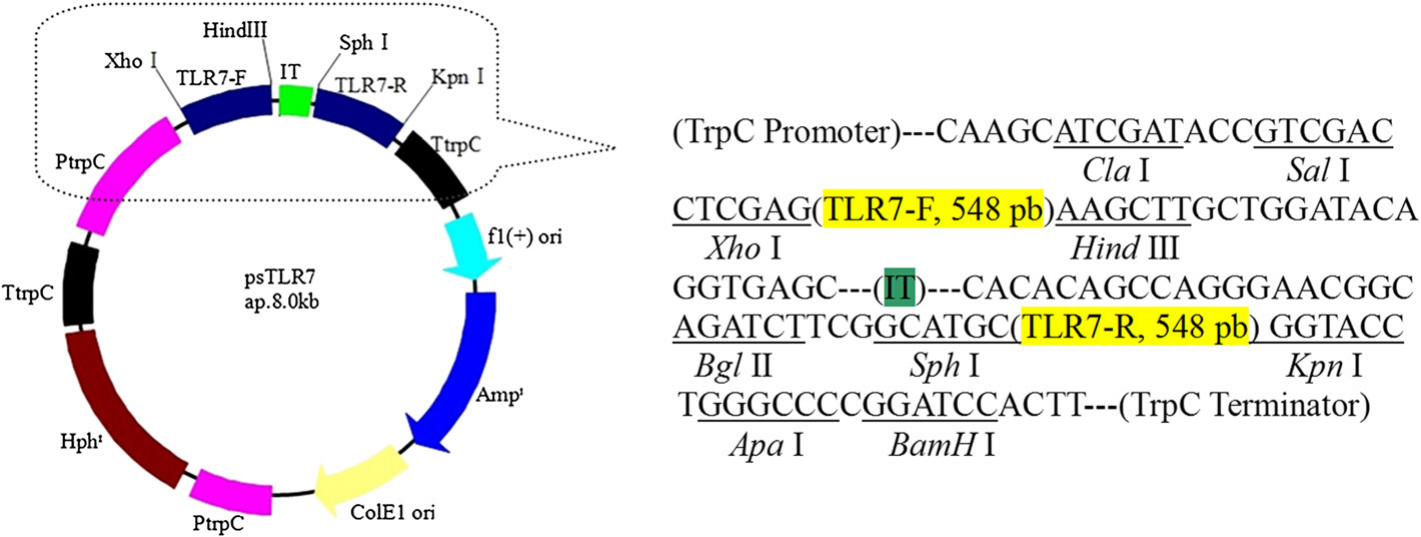
Construction of plasmid psTLR7. IT: intron; TLR7-F: forward fragment of TLR7 gene; TLR7-R: Reverse fragment of TLR7 gene; TtrpC: terminator; PtrpC: promoter; Amp^r^: Ampicillin resistance gene; Hph^r^: hygromycin B phosphotransferase resistance gene; XhoI, HindIII, SphIand KpnIare the site for cutting of DNA restrictive enzymes

**Fig. 2:**
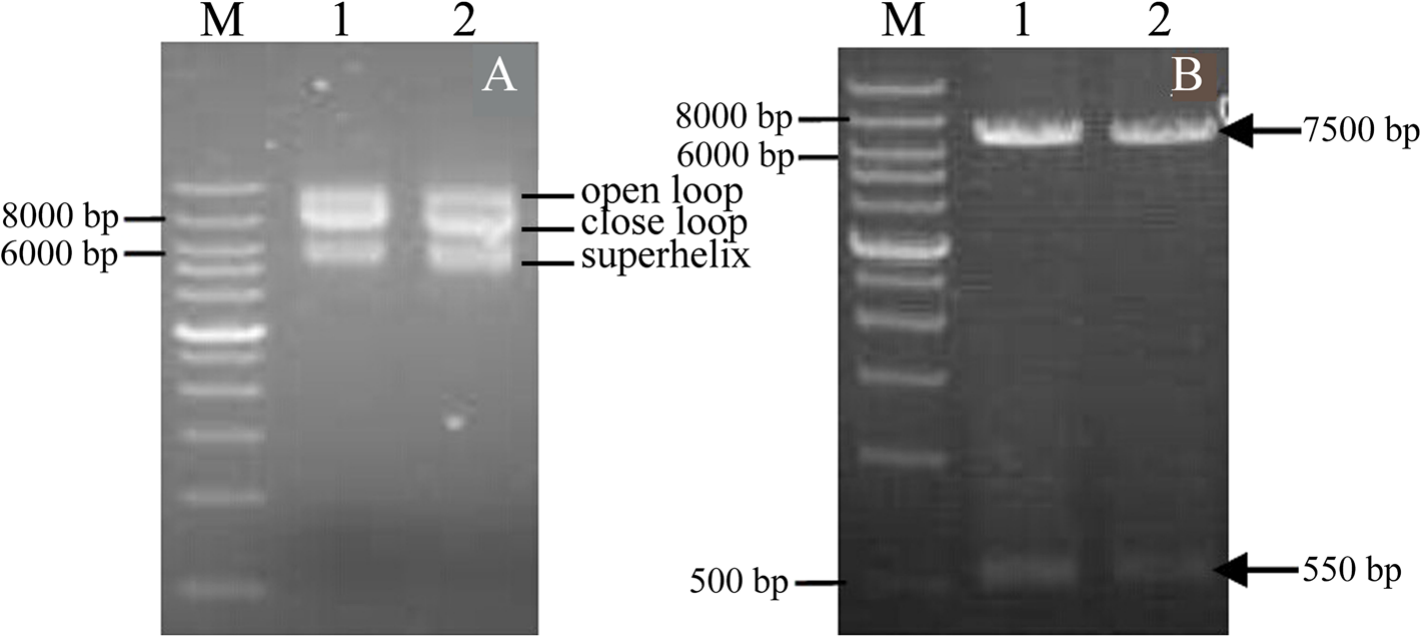
Electrophoresis profile of psTLR7. **a**: Electrophoresis of psTLR7. lane M, wide range DNA marker, lane 1–2, psTLR7 with the three conformations of open loop, close loop and superhelix. **b**: Double enzyme digestion of psTLR7. lane M, wide range DNA marker; lane 1, double digestion of psTLR7 by XhoI and HindIII; lane 2, double digestion of psTLR7 by SphI and KpnI

The dsRNA expression plasmid targeting TLR7 gene of whitefly was successfully constructed and was named as psTLR7 (Fig. 1). The electrophoretic results indicated that psTLR7 had the three conformations of open loop, close loop and superhelix (Fig. 2a) and had two hydrolysates of ~7500 pb and ~550 pb by double enzymes digestion of XhoI and HindIII or SphI and KpnI (Fig. 2b). The ~550 pb hydrolysate was then sequenced and validated its identity of target fragment (548 bp) (Additional file 2). The size of psTLR7 was about 8 kb, which corroborated the expected 6.9 kb of psilent-1 plus 548 bp each of forward and reverse TLR7 fragments. It was conformed that the expression cassette of hairpin loop dsRNA was successfully cloned into the psilent-1 plasmid. Subsequently, psTLR7 was introduced into the parent strain of *I. fumosorosea*, IfB01.

### Development of transgenic *I. fumosorosea* lines

**Fig. 3:**
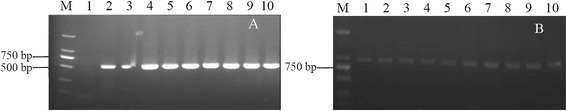
Electrophoresis profile of PCR products of IfB01-TLR7. **a**: PCR of target fragment; (**b**): PCR of hygromycin phosphotransferase gene. Lane M: DL2000 Marker, lane 1: plasmid pSilent-1, lane 2: psTLR7, lane 3–10: generation 1–8 of IfB01-TLR7. The objective fragment size was about 550 bp, while the hygromycin B phosphotransferase gene fragment about 850 bp

One stably inherited recombinant strain expressing dsRNA of the whitefly *TLR7* gene was selected and named as IfB01-TLR7. The strain showed good genetic stability. After transferring for eight generations, the strain still possessed the *TLR7* gene fragment (Fig. 3). The target fragment size was ~550 bp, while the *Hph*^*r*^ gene fragment was ~850 bp. The sequencing results indicated that the target fragment in IfB01-TLR7 was in agreement with the fragment of psTLR7.

**Fig. 4:**
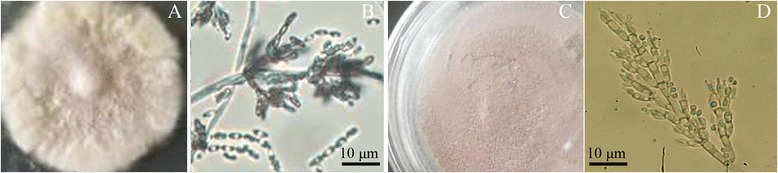
Morphological profile of strain IfB01 and IfB01-TLR7. **a**: the colony of strain IfB01 on Czapek Dox plate, (**b**): the sporulation structure and conidia of strain IfB01, **c**: the colony of strain IfB01-TLR7 on Czapek Dox plate, **d**: the sporulation structure and conidia of strain IfB01-TLR7

Although the morphological features of the parent and the recombinant strains were similar, the IfB01-TLR7 showed purple colored colonies and the IfB01 strain presented light yellow colonies. Additionally, the strain IfB01-TLR7 possessed shorter phialides and its conidia were different from the parent strain, IfB01 (Fig. 4).

### Virulence of IfB01-TLR7 and IfB01 strains against B-biotype *B. tabaci*

**Fig. 5:**
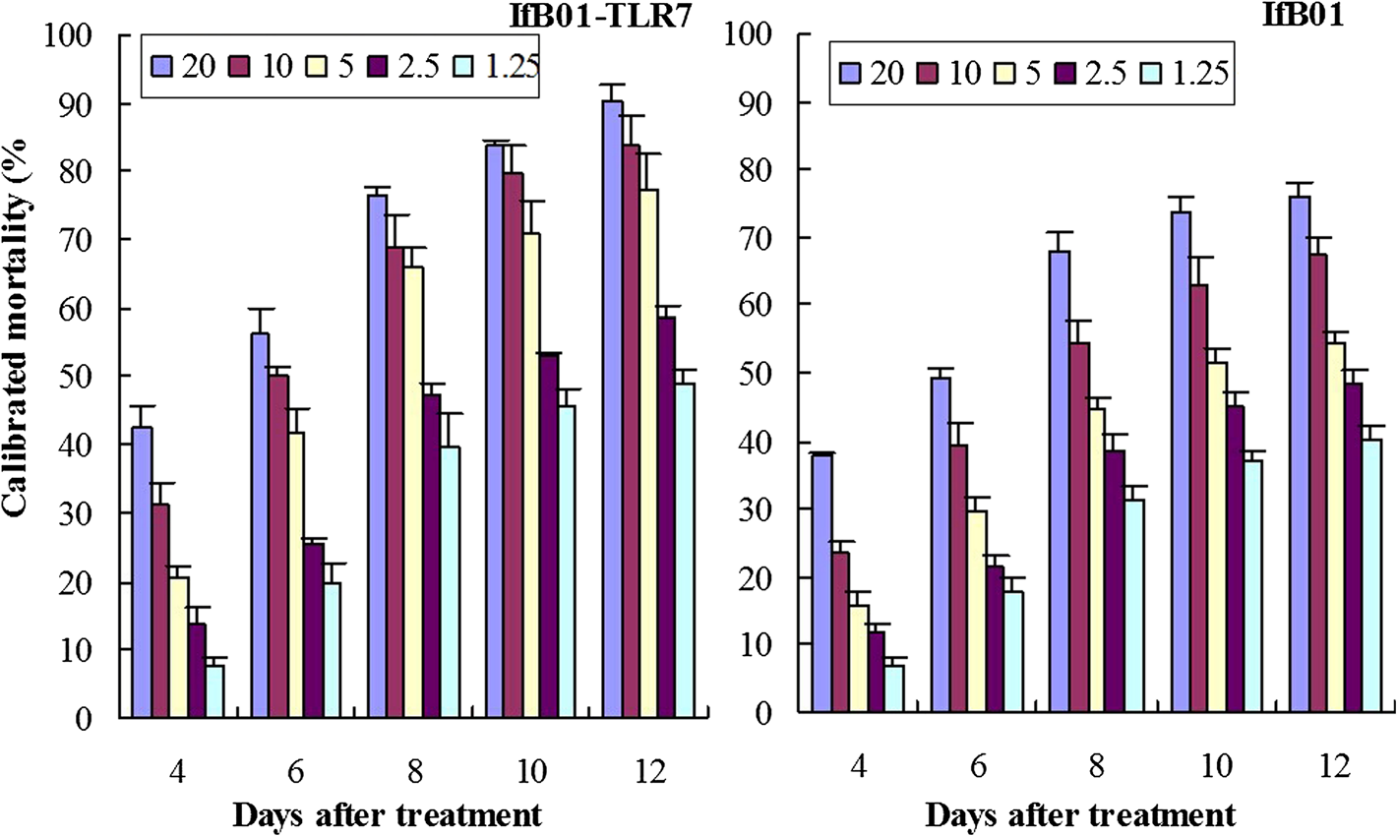
The mortalities of 2nd instar nymphs of B-biotype *Bemisia tabaci* treated with IfB01-TLR7 and IfB01 strain. The different shaded bars represent different spore concentrations (×10^6^ spores/mL), The error bars represent standard errors

The cumulative calibrated mortalities of whitefly were various in different treatments. Totally, the larger mortalities were observed in the treatments with higher concentrations of spores (Fig. 5). In comparison to the parent strain IfB01, the insecticidal effects of the recombinant strain IfB01-TLR7 were seen to improve evidently in different treatments. After 12 d of treatment at the concentration 2.0 × 10^7^ spores/mL, IfB01-TLR7 obtained a whitefly mortality of 90.33 % compared with 76.00 % of IfB01. Meanwhile, IfB01-TLR7 and IfB01 yielded 42.67 % and 37.67 % mortality rates, respectively, in the treatment with 1.25 × 10^6^ spores/mL on day 4.

The smaller LC_50_ values of IfB01-TLR7 indicated that the recombinant strain had higher virulence than the parent strain, IfB01 (Table 2). For instance, on the 8th day of post-treatment, IfB01-TLR7 revealed an LC_50_ value of 2.37 × 10^6^ spores/mL as compared to a value of 6.18 × 10^6^ spores/mL obtained by the IfB01 strain. In addition, treatment with the concentration of 5 × 10^6^ spores/mL yielded LT_50_ of 9.35 and 7.07 d in IfB01-TLR7 and IfB01 strains, respectively, indicating shorter periods of LT_50_ for IfB01-TLR7 (Table 3).

**Table 2:** Equations of LC-p and LC_50_s of fungal strain/dsRNA against whitefly

Strain		Intercept	Slope	*χ* ^2^	p	LC_50_ (95 % confidence interval) (×10^6^spores/mL)
IfB01	4d	−1.615	0.958	1.853	0.604	48.611 (33.454–83.933)
	6d	−1.061	0.789	0.753	0.861	22.083 (16.413–33.808)
	8d	−0.609	0.771	2.764	0.429	6.175 (5.063–7.618)
	10d	−0.451	0.797	1.372	0.712	3.681 (2.935–4.473)
	12d	−0.363	0.797	1.455	0.693	2.854 (2.196–3.516)
IfB01-TLR7	4d	−1.521	1.029	0.145	0.986	30.065 (22.751–44.145)
	6d	−0.920	0.878	4.035	0.258	11.137 (9.148–14.245)
	8d	−0.311	0.828	5.594	0.061	2.374 (1.532–3.150)
	10d	−0.328	1.088	5.071	0.079	2.004 (1.403–2.570)
	12d	-0.265	1.245	4.148	0.126	1.632 (1.141–2.101)

**Table 3:** Equations of LT-p and LT_50_s of fungal strain against whitefly

Strain (×10^6^spores/mL)		Intercept	Slope	*χ* ^2^	p	LT_50_ (95 % confidence interval) (d)
IfB01	10	−1.364	0.176	2.493	0.288	7.77 (7.35–8.24)
	5	−1.615	0.173	3.567	0.168	9.35 (8.81–10.05)
IfB01-TLR7	10	−1.327	0.220	1.459	0.482	6.04 (5.66–6.39)
	5	−1.659	0.235	12.204	0.002	7.07 (4.30–9.91)

### Validation of expression of the whitefly *TLR7* gene

**Fig. 6:**
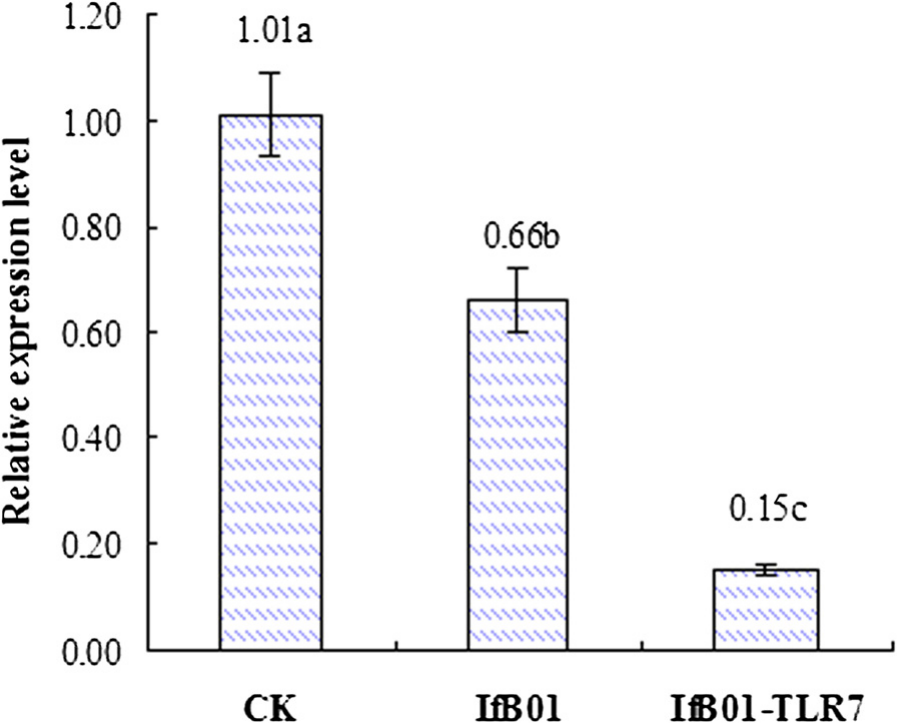
The relative expression level of TLR7 gene of whitefly infected by strain IfB01-TLR7 and IfB01

In the qPCR validation experiment, the relative expression level of the whitefly *TLR7* gene infected by the recombinant strain IfB01-TLR7 was recorded as 0.15, which was lower than that infected by the IfB01 parent strain (0.66) and the control (1.01) (Fig. 6).

## Discussion

RNAi is recognized as a promising new pest control technology, but there are several problems associated with introducing the exogenous dsRNA/siRNA into target pests, which restrict its practical application in the fields.

To the best of our knowledge, this is the first study wherein a recombinant *I. fumosorosea* strain (IfB01-TLR7) was constructed for the expression of the dsRNA targeting the immune-related gene, *TLR7* of B-biotype *B. tabaci*. The target gene, *TLR7*, was successfully knocked down after whitefly nymphs were infected with the IfB01-TLR7 strain. Meanwhile, the insecticidal activity of IfB01-TLR7 against whitefly was apparently increased. Our study have suggested that dsRNA can be delivered into target pests through infection of entomopathogenic fungus, and that the expression of specific dsRNA in entomopathogenic fungus may result in the development of a new RNAi methodology for pest control.

Although the virulence of our new genetically modified fungal strain was not extremely improved in comparison to the parent strain, the increase of more than 2-fold LC_50_ is still commendable for recombinant strain because the parent strain was selected from lots of wild strains and has excellent effectiveness to whitefly. What is the reason of the genetically modified strain? For the total insecticidal activity of modified strain, it is because of the combination of gene knock down and fungal infection. However, for the increased toxicity (LC_50_ decreased more than 2-fold), it should be contributed to gene knock down. In the future, it is maybe practicable that introduce two or more exogenous dsRNAs to get more effective genetically modified fungul strain.

The plasmid, pSilent-1, has been commonly used for silencing fungal genes [28]. However, there is no published report on the use of pSilent-1 in developing a transgenic entomogenous fungus strain in order to knock down the insect (host) target gene. The current experiment has, therefore, opened up a new avenue for the application of this gene silencing plasmid.

It is difficult to understand the mechanism of that the dsRNAs produced by cells of IfB01-TLR7 release and enter into the whitefly cells, because there are not sufficient information available. Many researchers have reported that entomogenous fungi penetrate the cuticle of the host and enter the hemocoel to morphologically transform from mycelia to blastospores (yeast-like hyphae). The type of blastospores benefits the fungus spread to whole body and adaptation for the hemolymph immunity [13, 14]. However, the insect host has effective innate immunity system to defend the invasion of fungus. Blastospores are attacked by hemocytes and humoral immunity. Generally, hemocytes damage and digest the blastospores by means of encapsulation, nodulation, and phagocytosis. In the process, the dsRNA may be released from the fungal cells and get absorbed by the hemocytes. This phenomenon may be considered as one of the mechanisms associated with the entry of specific dsRNA into the host cells. Secondly, we supposed that the fungal cells release the plasmid into the host hemocoel or other tissues. This might be attributed to the structure of the blastospore, which has a thick cell wall and hydrophilic cell surfaces [29]. Nevertheless, either of the speculated mechanisms requires further research to elucidate the course of entry of the specific dsRNA into the host cells.

There are still many aspects of this study, which remain unclear; like, how is the systemic RNA interference in host insect arisen. Therefore, further research is required to provide greater insights into the use of RNAi technology for the successful development of myco-insecticides and pest biocontrol.

## Conclusion

The expression of the dsRNA of whitefly TLR7 gene in recombinant *I. fumosorosea* strain successfully knocked down the host target gene by infecting the nymphs and enhanced the whiteflies mortality. The present study will give insight to new application of RNAi technology for more effective biocontrol of this pests.

## Additional files

Additional file 1:
**Unigene20701_B4592195 (TLR7 gene fragment) seguence.**
Additional file 2:
**The sequence of fragment in plasmid pSilent-1-TLR7F corresponding to whitefly’s TLR7 gene.**

## Abbreviations

Amp: Ampicillin
cDNA: Complementary DNA
ddH_2_O: Double distilled H2O
DNA: Deoxyribonucleic acid
dsRNA: Double-stranded ribonucleic Acid
*Hph*^*r*^: *Hygromycin B phosphotransferase* gene
LB: Luria-Bertani
mRNA: Messenger ribonucleic acid
PDA: Potato dextrose agar
PCR: Polymerase chain reaction
PEG: Polyethylene glycol
qPCR: Quantitative polymerase chain reaction
RNA: Ribonucleic acid
RNAi: Ribonucleic acid interference
siRNA: Small interfering RNA
Tris: Tris (Hydroxymethyl) aminomethane
*TLR7*: *Toll-like receptor 7*
