# Supplementary material for: Expression of dsRNA in recombinant Isaria fumosorosea strain targets the TLR7 gene in Bemisia tabaci
Source: BMC Biotechnol. 2015 Jul 22;15:64. doi: 10.1186/s12896-015-0170-8 (PMC4509747; doi:10.1186/s12896-015-0170-8)
Supplement: Additional file 1: — Unigene20701_B4592195 (TLR7 gene fragment) seguence. [file 12896_2015_170_MOESM1_ESM.doc]

Additional file 1. Unigene20701_B4592195 (TLR7 gene fragment) seguence

| 001  061  121  181  241  301  361  421  481  541  601  661  721  781  841  901  961  1021  1081  1141  1201  1261  1321  1381  1441  1501  1561  1621  1681 | TTAGTTTTGAATTTTTTAGCGTGCGCTCTGGCAAGCGAACCTTGCAACGTATTCCTCGAG  CCCAATGCAACCGCGCTTTGCAAACTCCCAACGATCCCCCGGGAGTTGATTCTGCCGGAG  AGCGATAATGTGATCAAACTCAGATTGGAGTGCAACCAGGATATCCACGCCGAAAGTTTC  ATCTACCTGAGCCTCCTCTCGCATTTCAACCAAATCGAAGAGTTCAACCTCACCAACTGC  AAAGTCGCCGAGATATCCGAGAATGTGTTCAGCCAGAGCCCCGGCCTCAAAAAGCTCACC  GTCAACTCTCGAAACTTCGACTGGTCACCGACGAAATCCCTGAGGATCAAGAGCAGGAGT  TTCCGACCTTTGAAGGAGCTCCATCACCTGGACCTGAGTTTCAATAACATGGACTCGCTG  CCGGACGGCGTGTTTTGCCCGTTGAAAAAGCTGCAGCATTTGAATTTATCCAACAACGCC  ATCGCAGATATAACAAGGCTGGGACTCTCGGCGAAGAAATGGGCCCCTCCTCGCGTGTCG  AGCCCGCGACCCACGACATCGGAGGGCGACACGGACGGGGAGGGCACCACCGAGTGCCAT  GGCGGCAGTGAACTTCGAACTTTAGACCTCTCGGCCAACCGCATCCAAAGCCTCGCCGAG  CTTTCCGACGTTTCGAAATTCAAGCGGCTCCACACCCTCCTTTTGAACGACAATCTTATC  ACCGAGATACCGAAAAACGCCTTCTCAAGCCTTCACAACGTGCATATCCTTAACCTCACA  AACAACAACATCCAAACTTTGACGGAGAACGTGTTCACCAACTGCAAAGAGCTTCAGGAA  ATCCATTTGCAGAATAATTCTCTGGCGCAACTGCCGAAGGGGCTGTTGAATCATTTAAAC  AAGCTTTTAATACTCGACTTATCATCGAATCAACTGACTTCCAATCAAATAGACGAGTCC  ACGTTCGTCGGCCTTATCAGGCTCATTGTCCTGAATCTCTCCAGCAACAGGCTGACGCGC  ATCAACTCCAAAACGTTCAAGGACTTGCTTTTCCTTCAGATCTTGGATTTACACAACAAC  TCGATCGGTTTCATCGAAAACGACTCGTTCCTGCCGTTGTACAACTTGCACACTTTGAAC  TTGAGCAATAATCGTCTGCACCACGTGACCTCCAATCTGCTCAACGGGTTGTTCGTTCTC  ACCAAGTTGAACTTGAACAATAATTTAATCACGACCGTCGATGAGATCGCCTTCAGGAAC  TGTTCCGATCTGAAAGAGCTTGACCTCAGCTCGAACGCCATCTCTGAGCTTCCAACGGCG  CTAACCGACCTGTCCTTCCTGAAGTCTCTCGATCTGGGCGAGAATCAGATCTCGGAGATT  AAACCGAACTCTTTTAAAAATATGGCCCAATTGACGGGGCTTCGGCTGATAGACAACAGC  ATAGGAAACTTGACGAAAGACATGTTCGTCCTCCAAGTTCTGAACTTGGCTAAGAATAAA  GTGCAATCGATCGAACGCGGGACTTTCGACAAGAACGTCCAGATCGAAGCCATCCGGTTG  GACTATAATTACTTGCAAGACATCAACGGCGTGTTTTCTAGCCTCGTGTCATTGCTATGG  CTGAATCTGTCTGATAATCATTTAGTTTGGTTCGATTACGCGTTCCTGCCGACGAATCTG  AAGTGGCTTGATATCCACAACAACTACATCGAGAAATTGGGTAACTATTACAAAATT |
| --- | --- |
