# Supplementary material for: Expression of dsRNA in recombinant Isaria fumosorosea strain targets the TLR7 gene in Bemisia tabaci
Source: BMC Biotechnol. 2015 Jul 22;15:64. doi: 10.1186/s12896-015-0170-8 (PMC4509747; doi:10.1186/s12896-015-0170-8)
Supplement: Additional file 2: — The sequence of fragment in plasmid pSilent-1-TLR7F corresponding to whitefly’s TLR7 gene. [file 12896_2015_170_MOESM2_ESM.doc]

Additional file 2. The sequence of fragment in plasmid pSilent-1-TLR7F corresponding to whitefly’s TLR7 gene

| 001  051  101  151  201  251  301  351  401  451  501 | CACGCCGAAAGTTTCATCTACCTGAGCCTCCTCTCGCATTTCAACCAAAT  CGAAGAGTTCAACCTCACCAACTGCAAAGTCGCCGAGATATCCGAGAATG  TGTTCAGCCAGAGCCCTGGCCTCAAAAAGCTCACCGTCAACTCTCGAAAC  TTCGACTGGTCACCGACGAAATCCCTGAGGATCAAGAGCAGGAGTTTCCG  ACCTTTGAAGGAGCTCCATCACCTGGACCTGAGTTTCAATAACATGGACT  CGCTGCCGGACGGCGTGTTTTGCCCGTTGAAAAAGCTGCAGCATTTGAAT  TTATCCAACAACGCCATCGCAGATATAACAAGGCTGGGACTCTCGGCGAA  GAAATGGGCCCCTCCTCGCGTGTCGAGCCCGCGACCCACGACATCGGAGG  GCGACACGGACGGGGAGGGCACCACCGAGTGCCATGGCGGCAGTGAACTT  CGAACTTTAGACCTCTCGGCCAACCGCATCCAAAGCCTCGCCGAGCTTTC  CGACGTTTCGAAATTCAAGCGGCTCCACACCCTCCTTTTGAACGACAA |
| --- | --- |
